# Carriers of mitochondrial DNA macrohaplogroup L3 basal lineages migrated back to Africa from Asia around 70,000 years ago

**DOI:** 10.1186/s12862-018-1211-4

**Published:** 2018-06-19

**Authors:** Vicente M. Cabrera, Patricia Marrero, Khaled K. Abu-Amero, Jose M. Larruga

**Affiliations:** 1 Departamento de Genética, Facultad de Biología, Universidad de La Laguna, E-38271 La Laguna, Tenerife, Spain; 2 Research Support General Service, E-38271, La Laguna, Tenerife, Spain; 3 Glaucoma Research Chair, Department of Ophthalmology, College of Medicine, King Saud University, Riyadh, Saudi Arabia; 4 Department of Ophthalmology and Visual Sciences, University of Illinois at Chicago, Chicago, IL USA

**Keywords:** Human evolution, Mitochondrial DNA, Haplogroup L3, Y-chromosome, Haplogroup E, Out-of-Africa

## Abstract

**Background:**

The main unequivocal conclusion after three decades of phylogeographic mtDNA studies is the African origin of all extant modern humans. In addition, a southern coastal route has been argued for to explain the Eurasian colonization of these African pioneers. Based on the age of macrohaplogroup L3, from which all maternal Eurasian and the majority of African lineages originated, the out-of-Africa event has been dated around 60-70 kya. On the opposite side, we have proposed a northern route through Central Asia across the Levant for that expansion and, consistent with the fossil record, we have dated it around 125 kya. To help bridge differences between the molecular and fossil record ages, in this article we assess the possibility that mtDNA macrohaplogroup L3 matured in Eurasia and returned to Africa as basal L3 lineages around 70 kya.

**Results:**

The coalescence ages of all Eurasian (M,N) and African (L3 ) lineages, both around 71 kya, are not significantly different. The oldest M and N Eurasian clades are found in southeastern Asia instead near of Africa as expected by the southern route hypothesis. The split of the Y-chromosome composite DE haplogroup is very similar to the age of mtDNA L3. An Eurasian origin and back migration to Africa has been proposed for the African Y-chromosome haplogroup E. Inside Africa, frequency distributions of maternal L3 and paternal E lineages are positively correlated. This correlation is not fully explained by geographic or ethnic affinities. This correlation rather seems to be the result of a joint and global replacement of the old autochthonous male and female African lineages by the new Eurasian incomers.

**Conclusions:**

These results are congruent with a model proposing an out-of-Africa migration into Asia, following a northern route, of early anatomically modern humans carrying pre-L3 mtDNA lineages around 125 kya, subsequent diversification of pre-L3 into the basal lineages of L3, a return to Africa of Eurasian fully modern humans around 70 kya carrying the basal L3 lineages and the subsequent diversification of Eurasian-remaining L3 lineages into the M and N lineages in the outside-of-Africa context, and a second Eurasian global expansion by 60 kya, most probably, out of southeast Asia. Climatic conditions and the presence of Neanderthals and other hominins might have played significant roles in these human movements. Moreover, recent studies based on ancient DNA and whole-genome sequencing are also compatible with this hypothesis.

**Electronic supplementary material:**

The online version of this article (10.1186/s12862-018-1211-4) contains supplementary material, which is available to authorized users.

## Background

Based on molecular genetics analyses, the hypothesis of a recent African origin of modern humans that occurred approximately 200 thousand years age (kya) was formulated three decades ago [1]. Today this hypothesis is widely accepted. There is also multidisciplinary agreement that the out-of-Africa expansion of modern humans promoted the extinction of other hominins in Eurasia with only a minor introgression of their genomes into modern human DNA [2]. However, despite the enormous quantity of data accumulated to date, mainly from the analysis of mtDNA and Y-chromosome haploid markers, there is a lack of consensus regarding the time(s) of modern human dispersal out of Africa and route(s) followed. All of the indigenous mtDNA diversity outside Africa is comprised into clades M and N, which are derivative branches of the African haplogroup L3 [3–5]. This fact places the genetic time frame for the out of Africa dispersal at approximately 55-70 kya, which is the coalescence age of haplogroup L3 [6]. Similarly, recent Y-chromosome sequence analysis detected a cluster of major non-African founder haplogroups originating within a short time interval at 47-52 kya [7]. However, estimates based on a molecular clock depend on the mutation rate employed [8, 9]. These temporal windows for the exit of modern humans from Africa conflict with fossil, archaeological and ancient DNA data on the timing of the initial migration of anatomically modern humans (AMHs) out of Africa. Skeletal remains unearthed in the Skhul and Qafzeh caves demonstrated that early modern humans were present in the Levant between 125 and 80 kya [10]. The discovery of modern human teeth in southern China dated to 120-80 kya [11], also supports the presence of AMHs in eastern Asia during this period. Several archaeological studies uncovered Middle Stone Age (MSA) lithic assemblages, dated at approximately 125-75 kya, in different regions of the Arabian Peninsula, presenting affinities with northeastern African assemblages of the same period [12–14]. These findings suggest that African AMHS may have extended their geographic range to eastern and northern Arabia, as well as south Asia, as early as 125 kya, long before the time frame of the migration that shaped the modern global genetic pool as suggested by molecular data.

Ancient DNA (aDNA) analysis is an important tool in the reconstruction of past human history. Based on aDNA analyses, the Neanderthal introgression into modern humans in Europe has been dated to within 35-65 kya [15], which is well within the molecular clock-based time frame established for the African exit of modern humans. However, an ancient gene flow from early modern humans into the ancestors of eastern Neanderthals more than 100 kya was recently reported [16]. These data evidenced that early modern humans and ancestors of Neanderthals from the Siberian Altai region interbred much earlier than previously thought. Furthermore, whole-genome based studies place the split of Eurasian from African populations at 88-112 kya [17], and the presence of AMHS out of Africa has been documented before 75 kya [18]. A way to reconcile these contradictory pieces of evidence is to state that all these ancient movements out of Africa, prior to 70 kya, did not significantly contribute genetically to present-day human populations [18]. However, major efforts should be dedicated to resolving the conflicting evidence.

Concerning the potential routes followed by modern humans out of Africa, there are two, non-mutually exclusive main alternatives: a northern dispersal along the Nile-Sinai corridor, and a southern dispersal from the Horn of Africa across the Bab al Mandeb Strait. In early mtDNA phylogeographic studies, the virtual absence of mtDNA haplogroup M in the Levant, and its presence in Ethiopia, southern Arabia, the Indian subcontinent and East Asia, rendered M the first genetic indicator of a southern-route exit from eastern Africa [19]. Shortly after these studies, based on the rarity of mtDNA haplogroup N(xR) in India, and its continuous presence above the Himalayas, we proposed an additional northern route through the Levant [4]. Since that time, intensive and extensive research on mtDNA has been carried out, on populations not only from Central [20–23] and East Asia [24–26] but also from the regions along the hypothetical southern route, such as India [27–30], mainland southeastern Asia [31–33], island southeast Asia [34–39], New Guinea, North Island, Melanesia and Australia [40–44]. The most unexpected results were that some N haplogroups in southern China (N10, N11) were older than the oldest N western Asia lineages (N1, N2), and that some M haplogroups in Melanesia (M27, Q) were older than the oldest Indian M lineages (M2, M33). Different researchers have provided conflicting interpretations of these results. Some perceived them as confirming a rapid, southern coastal spread of modern humans from Africa [30, 45–47]. Others postulate ancient local population differentiation in each region without any evidence of the shared ancestry expected by the southern dispersal model [48–50]. The existence of a northern route, deduced from the phylogeography of macrohaplogroup N [4], has received additional support from the fossil record [11], whole-genome studies comparing Egyptian and Ethiopian populations [51], and the fact that all non-African populations present a signal of Neanderthal introgression [52]. However, we realized that what actually macrohaplogroup N suggests is a human movement from southeastern Asia to western Asia [53]. We observed the same tendency for macrohaplogroup M, in this case, expanding westwards to India [54]. A similar trend was observed for macrohaplogroup R, the main sister branch of N [55]. Thus, we confirmed that macrohaplogroup M and N indicated, major southern and northern expansions, respectively, of modern humans but, in the opposite sense we had predicted previously [4]. Studies based on Y-chromosome sequences also suggested southeastern Asia as an early center of human expansions [56–59]. If one accepts that basal L3 lineages (M, N) evolved independently in southeastern Asia and not in Africa or near the borders of the African continent where the remaining L3 lineages expanded, one is confronted with the question of where the basal trunk of L3 evolved. A gravitating midpoint between eastern Africa and southeastern Asia would situate the origin of L3 in inner Asia, with possible opposite-direction expansions back to Africa and forward to eastern Asia. This possibility has been modeled, along with other options, by others obtaining the highest likelihood value among competing models [60]; however, it has not received the attention it deserves. The parallelism of this early back-to-Africa scenario of mtDNA haplogroup L3 with that proposed for the Y-chromosome haplogroup E [61] is striking.

In this work, we have improved the phylogeny of some African mtDNA L3 subclades. In addition, we have compared the frequency distribution of the younger African mtDNA haplogroup L3 and Y-chromosome haplogroup E in the main regions of the African continent. With these data at hand, we assess the possibility of the following scenario: L3 exited from Africa as a pre-L3 lineage that evolved as basal L3 in inner Asia. From there, it expanded, returning to Africa as well as expanding to southeastern Asia, giving rise to the African L3 branches in eastern Africa and the M and N L3 Eurasian branches in southeastern Asia, respectively. This model, which implies an earlier exit of modern humans out of Africa, has been tested against independent results from other disciplines.

## Methods

### Sampling information

A total of 69 complete mtDNA genomes were sequenced in this study (Additional file 1: Table S1). They comprise the main African L haplogroups, excepting L6. To remedy this lack, 12 previously published and complete L6 sequences were included in our phylogenetic tree (Additional file 2: Figure S1). The different branches of haplogroup L3 are represented by 45 of these sequences. To establish the relative frequency of mtDNA macrohaplogroup L3 in the main African regions, a total of 25,203 partial and total publicly available mtDNA sequences were screened. Of them, 1,138 represent our unpublished data (Additional file 1: Table S2). To establish the relative frequency of Y-chromosome macrohaplogroup E in the main African regions, a total of 21,286 publicly available Y-chromosome African samples were screened. Of them, 737 represent our unpublished data (Additional file 1: Table S2). All of our samples were collected in the Canary Islands or Saudi Arabia from academic and health-care centers. The procedure of human population sampling adhered to the tenets of the Declaration of Helsinki, and written consent was obtained from all participants before they participated in the study. The study underwent a formal review and was approved by the College of Medicine Ethical Committee of the King Saud University (proposal N° 09-659) and by the Ethics Committee for Human Research at the University of La Laguna (proposal NR157).

### MtDNA sequencing

Total DNA was isolated from buccal or blood samples using the POREGENE DNA isolation kit from Gentra Systems (Minneapolis, USA). The PCR conditions and procedures for sequencing the mtDNA genome were as previously published [4]. Successfully amplified products were sequenced for both complementary strands using the DYEnamic™ETDye terminator kit (Amersham Biosciences). Samples were run on MegaBACE™ 1000 sequencer (Amersham Biosciences) according to the manufacturer’s protocol. The 69 new complete mtDNA sequences have been deposited in GenBank under the accession numbers MF621062 to MF621130 (Additional file 1: Table S1).

### Compilation of Previously published data

Sequences belonging to specific mtDNA L haplogroups were obtained from public databases such as NCBI, MITOMAP, the-1000 Genomes Project and the literature. We searched for mtDNA lineages directly by using diagnostic SNPs (http://www.mitomap.org/foswiki/bin/view/MITOMAP/WebHome), or by submitting short fragments including the diagnostic SNPs to a BLAST search (http://blast.st-va.ncbi.nlm.nih.gov/Blast.cgi). Haplotypes extracted from the literature were transformed into sequences using the HaploSearch program (http://www.haplosite.com/haplosearch/process/) [62]. Sequences were manually aligned and compared to the rCRS [63] with the BioEdit Sequence Alignment program [64]. Haplogroup assignment was performed by hand, with screening for diagnostic positions or motifs at both hyper-variable and coding regions performed whenever possible. Sequence alignment and haplogroup assignment were carried out twice by two independent researchers and any discrepancy was resolved according to the PhyloTree database (Build 17; http://www.phylotree.org/) [65]. For the screening of the Y-chromosome haplogroup E, we considered samples as belonging to this haplogroup if they were found positive for, at least, the diagnostic DE-YAP or E-M40, E-M96 markers.

### Phylogenetic analysis

The phylogenetic tree was constructed by means of the Network program, v4.6.1.2, using, the Reduced Median algorithm, Median Joining algorithm and Steiner (MP) algorithm in sequential order [66]. Remaining reticulations were manually resolved. Haplogroup branches were named following the nomenclature proposed by the PhyloTree database [65]. Our coalescence ages were estimated by using statistics rho [67] and Sigma [68] and the calibration rate proposed by Soares et al. [6].

To calculate the total mean age of each haplogroup, we compiled all of the different estimation ages from the literature without taking into account the mtDNA sequence segment analyzed, the mutation rate considered, or the observed partial overlapping of the samples used. In cases where the same sample set was used to calculate its age by using multiple methods, we selected the age calculated based on the rho statistic as the most generalized method (Additional file 1: Table S3). To calculate haplogroup mean coalescence ages for the non-recombining region of the Y-chromosome (NRY), we compiled estimations preferably based on single nucleotide polymorphisms (SNPs) obtained by sequencing. When different mutation rates were used in the same study, we chose the age calculated based on the slowest mutation rate (Additional file 1: Table S4).

### Phylogeographic analysis

In this study, we focus on the earliest periods of the out-of-Africa spread of modern humans and the likely return to Africa of the carriers of primary mtDNA L3 and Y-chromosome E lineages.

As the phylogeography of the different branches of these lineages has been extensively studied by other authors to reveal more recent human movements on the continent, we focus here on the continental distributions in the major African regions. For phylogeographic purposes, we divided the African continent into the following eight major regions: 1. Northwest Africa (including Morocco, West Sahara, Algeria and Tunisia), 2. Northeast Africa (including Libya and Egypt), 3. West Sahel (including Mauritania, Mali, and Niger), 4. East Sahel (including Chad, Sudan, Ethiopia, Somalia, and Eritrea), 5. West Guinea (including Senegambia, Guinea-Bissau, Guinea-Conakry, Sierra-Leona, Liberia, Ivory-Coast, Burkina-Faso, Ghana, Togo, Benin, and Nigeria), 6. Central Africa (including Cameroon, Central African Republic (CAR), Congo Democratic Republic (CDR), Congo-Brazzaville, Gabon and Equatorial Guinea), 7. East Guinea (including Uganda, Rwanda, Kenya, and Tanzania), and 8. Southern Africa (including Angola, Zambia, Malawi, Mozambique, Zimbabwe, Botswana, Namibia and South African Republic (SAR)).

To evaluate the level of geographic structure in the mtDNA macrohaplogroup L3 and the Y-chromosome macrohaplogroup E in Africa, we performed AMOVA and K-means clustering analyses. We used the GenAlEx6.5 software to implement AMOVA and XLSTAT statistical software to perform the K-means clustering analysis. The possible associations between the frequencies of mtDNA macrohaplogroup L3 and those of the Y-chromosome macrohaplogroup E, for both the whole African continent and each of its principal geographic subdivisions were tested by Pearson correlation analyses using XLSTAT software. As an extensive overlap exists among the expansion ages of the L3 branches with those of the widespread African mtDNA macrohaplogroup L2, the global frequencies of L2 were included in the majority of the phylogeographic analyses performed.

## Results and discussion

### Phylogeny and affinities of our African complete sequences

Overall, our 69 mtDNA complete sequences (Additional file 2: Figure S1) could be allocated into previously defined clades in the PhyloTree database. Their closest affinities were with other sequences of the same haplogroups. Thus, our Kenyan L3a1a (Kn028) sequence shares tip mutations 514, 3796 and 4733 with a Tanzanian sequence (EF184630) but only mutation 514 with a Somalian sequence (JN655813) of the same clade. The Sudanese L3b1a (Su238) sequence shares the very conservative transition at 12557 with an L3b sequence (KF055324) from an African-American glaucoma patient [69]. Our L3b1a2 (Su002) sequence has matches at 195, 12490 and 16311 with several African sequences (EU092669, EU092744, EU092795, EU092825, EU9355449) with which it composes a new branch, L3b1a2a, defined by these three transitions. Similarly, the L3f2a1 (Su004) sequence has matches at mutated positions 6182, 8676, 9731, 12280, 12354 and 13105 with other published Senegalese sequences (JN655832, JN655841) with which composes a new derived branch. We expected L sequences detected in the Canary Islands to have their closest relatives among sequences from the African continent. This was observed in some cases; for example, the L3d1b3 (Go764) sequence from La Gomera island shares tip transitions 14040 and 16256 with an Ovimbundu isolate (KJ185837) from Angola [70]. However, unexpectedly, the Canarian sequence TF0005, allocated to the L3f1b subclade, has its closest relatives in the Iberian Peninsula, sharing the 8994 transition with two Asturian L3f1b sequences (KJ959229, KJ959230) [71]. Furthermore, the L3x2 (TF116) sequence from Tenerife shares all of its terminal variants(650, 7933, 8158, 15519, 16261) with sequences from Galicia (HQ675033, JN214446) and Andalusia (KT819228), not with African sequences. Saudi Arabia has been identified as an important receptor of mtDNA Eurasian lineages, as well as those of African origin. Arab sequences belonging to the L3i1a (AR429) and L3x1a1 (AR260) haplogroups have their closest relatives with sequences JN655780 and DQ341067, respectively, from nearby Ethiopia, and the L3h1b1 (AR381) sequence is identical to a previously published Yemeni isolate (KM986547). However, the L3h1b2 (AR221) sequence is most related to the JQ044990 lineage from Burkina Faso [72], with which it shares particular transitions at positions 7424, 13194, 16192 and 16218. The affinities of the Arab L1c2b1a'b (AR1252) with other sequences are the most unexpected. This sequence, particularly characterized by the presence of an insertion of 11 nucleotides at the 16029 position in the control region, has an exact match with an L1 isolate from the Dominican Republic (DQ341059). Its closest relatives in Africa, while lacking the above-mentioned insertion, are found in Angola (KJ185814) and Zambia (KJ185662) among Bantu-speakers [70]. The control region of this AR1252 isolate was previously published (KP960821). Concerning the less frequent L4, L5, and L6 clades, our L4b1a (Iv136) sequence from the Ivory Coast shares tip mutations 789, 7166 and 14935 with geographically nearby sequences (JQ044848, JQ045081) from Burkina Faso [72]. Similarly, the Arab L4a2 (AR1116) sequence is closely related to other African L4a2 sequences (EU092799, EU092800), and the L4b2a1 (AR197) isolate is identical to a sequence (KM986608) from Yemen [73]. From the analysis of partial sequences [53, 74], we can be certain that representatives of branches L4a1, L4a2 and L4b2 exist in Saudi Arabia. However, we have not yet detected sequences belonging to the large Sudanese L4b1b clade (Additional file 2: Figure S1). Published [53, 74] and unpublished data allow us to confirm that L5a is represented in Saudi Arabia by at least one lineage that has the following haplotype in/nearby the coding region: 15884, 16093, 16129, 16148, 16166, 16187, 16189, 16223, 16265C, 16311, 16355, 16362/ 73, 152, 182, 195, 198, 247, 263, 315iC, 455i2T, 459iC, 513, 522dCA, 709, 750, 769, 825A, 851, and 930. It represents 0.58% of the Saudi mtDNA gene pool. There is also a L5b lineage characterized by mutations at 15927, 16111, 16129, 16148, 16166, 16187, 16189, 16223, 16233, 16254, 16265C, 16278, 16360, 16519/ 73, 195, 247, 249d, 263, 315iC, 459iC, 501, 535, 750, 769, and 825A, with minor presence (0.09%) in Saudi Arabia. Furthermore, although we lack complete L6 sequences, we can confirm based on partial sequencing and specific SNP analysis, that L6a1 (0.13%) and L6b (0.09%) lineages are also present in the Saudi population [53, 74]. With 12 complete sequences, L3h is the best-represented haplogroup in our phylogeny (Additional file 2: Figure S1). In it, we have provisionally defined some new branches as follows: The retromutation at position 16223 defines a Sudanese L3h1a1a branch. Two clades, L3h1a2a1a and L3h1a2a1b are characterized by transitions 3892, 7705, and 15346 and transitions 5108 and 16165, respectively. An additional subclade, defined by transitions 7310, 13153, 14407 and transversion 9824A is provisionally named L3h1a2b1. In addition, after introducing the AR221 sequence, the old branch L3h1b2 is characterized only by transitions 294, 8842, 9758, 12882, 13437, 16129, and 16362.

Our only discrepancy with the PhyloTree phylogeny, regards the rare and old L5 clade. We have identified a new branch, provisionally named L5c (Additional file 2: Figure S1). In light of the information provided by this lineage, the PhyloTree L5b node, that joints haplogroups L5b1 and L5b2 by sharing retromutations at positions 182, 13105 and 16311 and a transition at position 16254 appears to lack phylogenetic robustness. The PhyloTree L5b2 clade is more appropriatelly considered a sister branch of our new Sudanese L5c sequence (Su412), which share a retromutation at the 195 position and transitions at the 6527 and 11809 positions.

Despite the small sample sizes employed, our coalescence age results fall within the standard deviations calculated for the different haplogroups (Additional file 1: Table S3). However, the mean age value for the L3 macrohaplogroup in Africa (71 ± 12 kya), which theoretically marks the upper-bound time for the out-of-Africa movement of modern humans, falls short compared to those estimated from the fossil record in the Levant [75].

### The Eurasian origin of mtDNA macrohaplogroup L3

The southern route hypothesis proposes that the Eurasian branches (M and N) of macrohaplogroup L3 differentiated in or near the African continent and rapidly spread across the Asian peninsulas to reach Australia and Melanesia [45]. Under this hypothesis, it is expected that, the coalescence ages of haplogroups should generally decrease from Africa to Australia. However, we have demonstrated that this is not the case [53–55]. Just on the contrary, the oldest M and N haplogroups are from southern China and Australasia, not India, and the associations between longitudinal geographic distances and relative ages of the M and N haplogroups run, contrary to expectation, from east to west [53, 54]. This presents us with a dilemma: It appears that two gravity centers of L3 expansion exist, one in Africa and one in southeastern Asia. A geographic equidistant midpoint would situate the primary radiation of L3 in India if a southern route were followed by the African colonizers and above the Himalayas, between Tibet and Pamir, if the northern route was followed. Furthermore, as the coalescence age of the African L3 branches and that of the Eurasian L3 (MN) branches are very similar (Table 1), at approximately 71 kya, the temporal and spatial midpoints might also coincide. As the group of modern humans that hypothetically returned to Africa is expected to include both females and males, searching for Y-chromosome phylogenetic and phylogeographic information might provide additional information.

**Table 1:** MtDNA and NRY mean values for MRCA, Out-of-Africa and haplogroup coalescences

Marker	MRCA	Out-of-Africa	Haplogroup splits
MtDNA	184 ± 61.0	71.0 ± 12.0^a^	L3'4'6: 95.8 ± 14.0
		71.0 ± 8.0^b^	L3'4: 84.1 ± 8.6
NRY	171.5 ± 13.7	93.9 ± 25.3	DE: 69.0 ± 6.7
			E: 65.5 ± 8.5

^a^L3 Africa; ^b^L3 Eurasia

### The Eurasian origin of Y-chromosome haplogroup E

An origin in Asia and return to Africa was proposed, long ago, for the Y-chromosome African haplogroup E [61]. This hypothesis was based on the derived state of its African YAP^+^ haplotypes 4 and 5 (haplogroup E) with respect to the ancestral Asian YAP^+^ haplotype 3 (haplogroup D). The later discovery of new markers evidenced that D and E were sister branches of the YAP^+^ node. Haplogroup D showed the derived status for M174 and the ancestral status for M40 in Asia, whereas haplogroup E was characterized by the derived status for M40 and the M174 ancestral status throughout Africa, thus the migratory sense between continents of both haplogroups could not be assured [76]. A few YAP^+^ individuals, ancestral for both markers, were detected in West Africa [77] and in Tibet [78]. Although assigned to the para-haplogroup DE*, its actual ancestral state could not be confirmed. Furthermore, a new mutation (P143) united the two other Eurasian haplogroups C and F as brothers and, in turn, DE and CF were joined to an ancestral node defined by mutations M168 and M294 [79]. The initial scenario proposed to explain this complicated situation was that two independent migrations out of Africa occurred: one marked by D, and the other marked by the CF pair of lineages [80]. However, a new interpretation arose following the discovery of more than 60,000 single nucleotide variants by next generation sequencing techniques. The most parsimonious interpretation of the Y-chromosome phylogeny constructed with these variants is that the predominant African haplogroup E arose outside the continent and back-migrated to Africa [59]. The DE split as a lower bound (69.0 ± 14.7 kya) and the radiation of E into Africa as an upper bound (65.5 ± 8.5 kya) yields a time frame highly coincidental with the dates estimated for the mtDNA haplogroup L3 expansions (Table 1). Furthermore, the spatial distribution of the residual Y-chromosome haplogroup D in Asia is a good indicator of the geographical location of the putative DE split. The highest frequency and diversity of D are in the Tibet region. Although D is also present at low incidence throughout southeast Asia, two other centers with notable frequency are in Japan and the Andaman Islands [78, 81, 82], possibly representing edge relic areas of a wider distribution long ago. There are no native D lineages in India, decreasing the possibility that this subcontinent was the center of the DE partition, which argues against a southern route. It is likely that the divergence of the Y-chromosome D and E haplogroups occurred up to the Himalayas and in or westward of the Tibet which coincides with the hypothetical bifurcation center proposed for the mtDNA L3 macrohaplogroup.

### Coetaneous east and westward expansions of modern humans from Central Asia

**Fig. 1:**
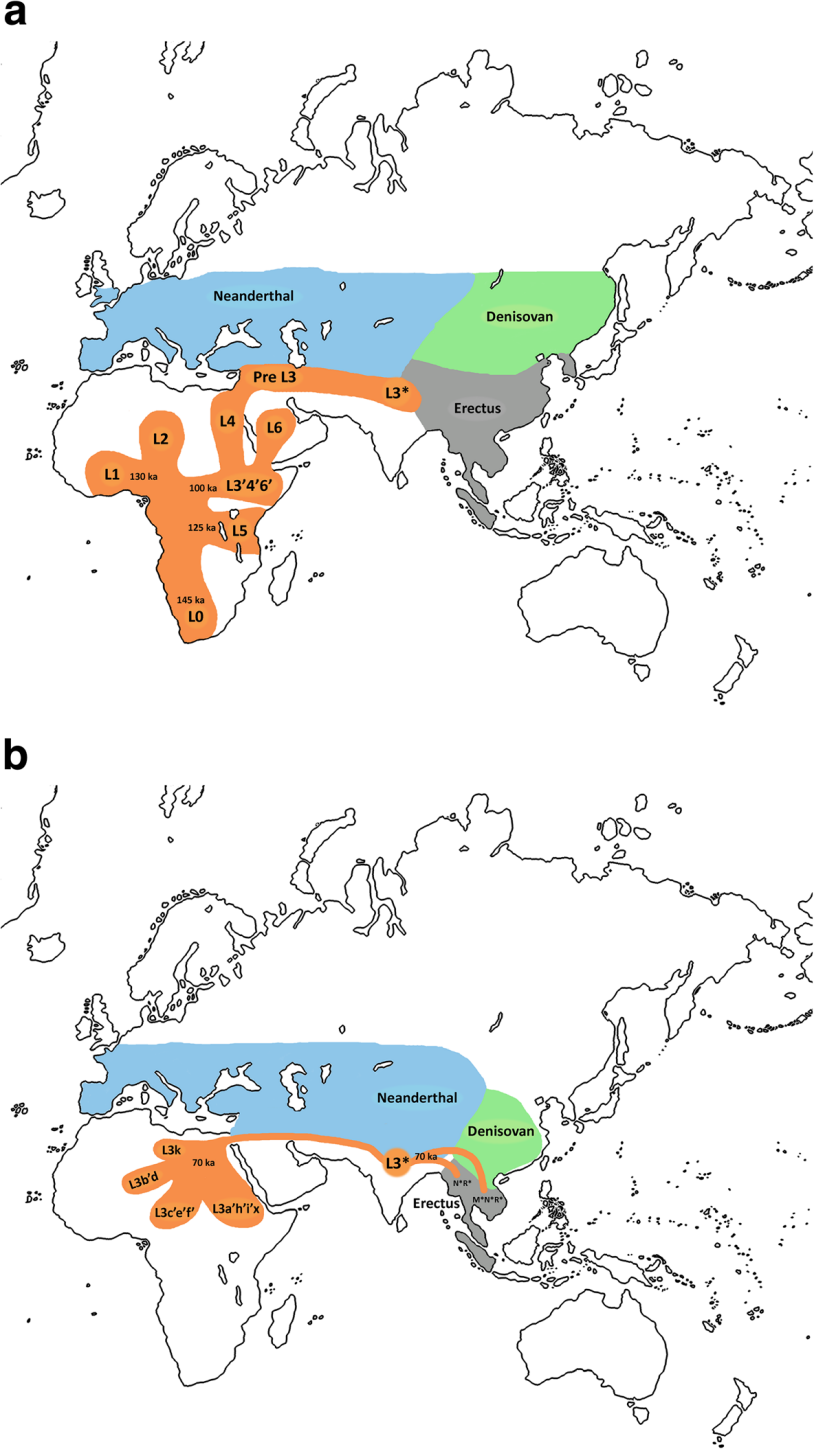
Geographic origin and dispersion of mtDNA L haplogroups: **a** Sequential expansion of L haplogroups inside Africa and exit of the L3 precursor to Eurasia. **b** Return to Africa and expansion to Asia of basal L3 lineages with subsequent differentiation in both continents. The geographic ranges of Neanderthals, Denisovans and Erectus are estimates only

The above described coincidental divergences of female and male lineages occurred during a glaciation period (70 - 100 Kya), it is possible that cold climatic conditions forced humans southwards and that, upon being confronted with the Himalayas, humans dispersed across southeastern and southwestern Asia. It is likely that this climatic change also led the Neanderthals to extend their southern range and, consequently increase their geographic overlap with humans and, possibly, Denisovans, outcompeting them in the search for resources (Figure 1). This southward retreat was more extensive in the west, as evidenced by the total occupation of the Levant by the Neanderthals approximately 70 kya [83] and the forced return of modern humans carrying the mtDNA L3 and Y-chromosome E basal lineages to Africa. However, 20 ky later the situation changed. Modern humans advanced westwards from inner Asia, displacing Neanderthals along the way and colonizing East Asia, South Asia, and Central Asia, from where they reached the Levant approximately 50 kya [84] and Europe shortly thereafter [85–87]. This westward colonization of modern human was also proposed based on an archaeological perspective [87, 88]. Under the proposed model, early modern humans would have left Africa much earlier than the time frame proposed by geneticists under mitochondrial molecular clock restrictions [89]. In an attempt to resolve this discrepancy, one could fix this period at the L3'4 or L3'4'6 mtDNA coalescence node, i.e., at approximately 80 to 100 kya (Table 1), such that mutations 769, 1018 and 16311, which define the basal L3* lineage, would have arisen after the movement out of Africa. Accordingly, the exit of the companion men could be dated at the split of branch CDEF-M168 from B-M181 approximately 86-120 kya [59, 90]. However, given the inaccuracies of the molecular clock, we prefer to rely on fossil and climatic records to establish the timing of the out-of-Africa movement of early modern humans across the Levant, which yields approximately 125 kya as the most likely period.

### The phylogeography of the L3 and E lineages inside Africa

The continental mean frequencies of the mtDNA and NRY haplogroups in the six main regions of the African continent are presented in Table 2. The mtDNA haplogroup L3 is more frequent in sub-Saharan Africa than in North Africa or the Sahel. In contrast, the Eurasian mtDNA haplogroups, including M1 and U6, are more frequent in northern Africa and the Sahel than in sub-Saharan Africa. In general, the Y-chromosome haplogroup E is more frequent in western than eastern Africa, while the Eurasian Y haplogroups show the opposite trend. This geographic distribution confirms that the history of Africa is marked by multiple Eurasian migratory waves that brought the first carriers of female haplogroup L3* and male haplogroup E* basal lineages into Africa. It has been suggested that the few sub-Saharan haplogroups present in northern Africa are the result of recent historical incorporations [91]. Studies of ancient DNA in the area appear to confirm this scenario both in the northwest [92] and the northeast [93]. The fact that L3k, the only autochthonous L3 lineage in northern Africa, has only a residual presence in the area also supports this scenario [94]. Under this supposition, male E lineages, currently present in North Africa, would have reached the area as a secondary wave accompanying Eurasian female lineages as M1 and U6. The main indigenous E clades in the region, E-M81 in the northwest and E-M78 in the northeast, are derived from haplogroup E-M35, which also includes the European Mediterranean and western Asian branches E-V13 and E-V22 [95]. In conflict with earlier studies that considered a Paleolithic implantation of E-M81 in the Maghreb [96, 97], later work suggested that the low microsatellite diversity of this clade in northwest Africa is better explained as the result of Neolithic or post-Neolithic gene flow episodes from the Near East [98]. However, the subsequent discovery of a new sister branch of E-M81, named E-V257 [99], without Near Eastern roots but present in the European western and central Mediterranean shores and in Cameroon and Kenyan populations [99, 100], weakened support for the suggested Levantine connection. Furthermore, E-M81 and E-M78 precursors are very old lineages that accumulated 23 and 16 mutations, respectively, in their basal branches. It has been reported that E-M78 radiated in eastern Africa between 20 and 15 kya while E-M81 did not, most likely because it was already in the Maghreb at that time. This timing coincides with the expansion age of the mtDNA U6a haplogroup in the area [101]. Thus, a recent re-expansion after a large bottleneck is the best explanation for the low variance of E-M81 in present day [102]. The persistence of an even older male demographic substrate in this area was evidenced by the detection of representatives of the deepest Y phylogenetic clades A0 and A1a in the region [103]. There is general consensus in attributing an eastern African origin to the initial expansion of the NRY haplogroup E in the continent [100]. Curiously, ancestral E* lineages have been detected in the Arabian Peninsula [104] and the Levant [105]. Regardless of its origin, haplogroup E shows lower frequencies in northeastern Africa and the eastern Sahel than in the west. The opposite pattern is observed in the frequencies of Y Eurasian haplogroups in the same areas (Table 2), which suggests later stronger Eurasian male gene flow throughout the northeastern side.

**Table 2:** MtDNA and Y-Chromosome mean haplogroup frequencies in the major regions of the African continent

Haplogroup	NW Africa	NE Africa	W Sahel	E Sahel	W Guinea	C Africa	East Guinea	S. Africa
mt-L0	0.90±0.88	2.50±1.00	1.52±0.91	7.56±3.94	2.27±3.03	4.35±2.10	30.55±6.58	56.5±8.03
mt-L1	5.01±1.31	2.72±0.62	17.83±2.90	3.47±2.77	15.78±7.30	34.85±6.55	4.72±3.14	2.13±0.50
mt-L2	7.96±6.37	6.03±3.28	30.88±15.52	19.07±12.91	36.21±11.26	22.35±12.99	10.70±6.99	16.15±16.17
mt-L3	12.06±7.05	13.93±5.74	31.85±17.83	27.06±12.64	36.82±10.66	33.10±17.51	35.59±11.57	23.84±18.80
mt-L4	0.24±0.14	1.34±0.21	0	4.94±3.19	0	0.20±1.25	9.14±4.78	0.57±0.31
mt-L5	0.02±0.04	0.49±0.68	0	4.23±3.54	0	0.10±1.30	2.47±1.84	0.45±0.23
mt-L6	0.04±0.09	0.08±0.12	0	0.91±1.41	0	0	0.27±0.38	0
mtMN	73.77± 10.88	72.91±7.70	17.92±5.02	32.76±4.56	8.92±3.34	5.05±3.13	6.56±4.73	0.36±0.28
Y-A	0.09±0.15	0.68±0.95	0	10.72±4.73	1.18±1.85	0.59±0.42	7.95±8.54	8.45±2.90
Y-B	0.34±0.31	0	0	8.96±13.18	2.14±3.23	7.23±3.48	17.48±10.97	10.70±3.34
Y-E	76.65±15.46	49.64±17.76	84.10±13.72	51.01±23.12	92.76±6.46	79.16±22.13	73.24±20.48	69.51±25.70
Y-F	22.92±7.35	49.68±8.00	15.90±5.36	29.31±6.08	3.92±2.87	13.02±3.44	1.33±1.21	11.34±4.12

Detailed frequencies for mtDNA haplogroups L2 and L3 and Y-chromosome haplogroup E, all around the African continent, are listed in Additional file 1: Table S2. We have included L2 in the analysis because it is the sister clade of the composite eastern African node L3'4'6 that, through consecutive range expansions, promoted the exit of the L3 precursor out of Africa. In addition, within Africa, several L2-derived spreads coincide in time with the later expansion of L3 branches in the continent. Furthermore, there is a suggestive positive association between the mean frequency estimates for L2 and the Y-chromosome haplogroup E across the major African regions (Table 2). In fact, there are significant positive correlations between E haplogroup frequency and both L3 (r = 0.400; *p* < 0.0001) and L2 (r = 0.347; *p* < 0.0001) mtDNA haplogroup frequency across Africa. An even stronger correlation is observed between E haplogroup frequency and the combined frequency of the two L2 and L3 haplogroup (r = 0.477; *p* < 0.0001). However, the strength of the association varies among the different regions considered, with no significant correlation in northern Africa, as can be expected from the discussion above. The correlation is barely significant in the Sahel region (r = 0.246; *p* = 0.045), but is highly significant in the remaining regions, particularly in southern Africa (r= 0.615; *p* < 0.0001). Nevertheless, these correlations are only slightly associated with geography, as indicated by the AMOVA results showing that only 4% of the variance is explained by differences among regions (Table 3). It appears that the E and L2/L3 expansions were strongest in western Sahel and western Guinea, where they replaced the majority of the oldest mtDNA (L0 and L1) and Y-chromosome (A and B) lineages (Table 2). We applied the k-means clustering algorithm to our L3 and E frequency data (Additional file 1: Table S2). The consecutive partitioning of the samples into clusters functions to minimize the variance within groups while augmenting the variance among them (Table 3). At k = 5, less than 20% of the variance is due to differences within clusters. At this value of k, the five classes obtained have centroid means for L3 and E that minimize the mean-square distance of the samples grouped to this center (Table 4 and Additional file 1: Tables S5, S6 and S7). Class I, characterized by relatively low frequencies for both the L3 and E haplogroups, encompasses the majority of the Khoesan-speaking groups from South Africa, Namibia, and Angola and the Hadza from Tanzania, as well as several pygmy groups from Cameroon, Gabon, CAR and Congo, such as the Baka and the Babinga. In addition to having different geographic locations, these groups are differentiated by the frequencies of other haplogroups. Thus, samples from Khoesan-speaking groups harbor high frequencies of mtDNA L0d and L0k haplogroups and Y-chromosome A lineages, while the Central African pygmies are characterized by the highest frequencies of mtDNA L1c and Y-chromosome B-M60 lineages. The Hadza share with pygmy groups high percentages of B-M60 chromosomes and the highest frequency of mtDNA haplogroup L4 among the entire African continent. Other groups that belong to this class are the majority of Nilotes from Sudan and Uganda, characterized by their high frequencies of mtDNA haplogroup L2a1, and samples from several Afro-Asiatic-speaking groups from Egypt and Sudan which also show high levels of L2a1 lineages and have many mtDNA L0a1 and Y-chromosome B representatives. The remaining of the samples from Khoesan-speaking groups fall into Class II having a low frequency of L3 and an intermediate frequency of E (Table 4), suggesting male-biased gene flow from western sub-Saharan Africans. This class also contains the remaining central Africa pygmies, including Sanga, Mbenzele, Biaka, and Mbuti, which all harbor high frequencies of Y B-M60 and L1c lineages. This class includes mainly northeast African and Ethiopian samples of individuals speaking Afro-Asiatic languages; some Nilo-Saharan-speaking groups such as the Fur from Sudan, the Anuak from Ethiopia and the Maasai from Kenya; and Bantu-speakers such as the Shona from Zimbabwe and Botswana and the Tswana from Botswana and South Africa. However, most of the northwestern African Afro-Asiatic speaking groups fall into Class III, defined by low frequencies of L3 and high frequencies of E (Table 4). A general low frequency of mtDNA haplogroup L2 is also a characteristic of this class. The majority of Berber and Tuareg samples belong to this class, including the Gossi, Tamashek, and Douentza from Mali and the Gorom from Burkina Faso. Interestingly, the click-speaking Sandawe and the Nilo-Saharan speaking Datog from Tanzania also belong to Class III. On the maternal side, these Tanzanian samples are characterized by relatively high frequencies of haplogroups L0a and L4. However, the most abundant components of this class are samples from Niger-Congo speakers, including the majority of the Senegalese samples as well as southern African-specific-Bantu-speakers such as the Zulu and Xhosa. Curiously, the click-speaking Xeg Khoesan and Khwe belong to this cluster, indicating substantial gene flow from Bantu-speaking immigrants. Such flow was reported long ago for the Khwe, who were found to be more closely linked to non-Khoesan-speaking Bantu populations [106]. Another class dominated by Niger-Congo speakers is Class IV, the largest class. It includes samples that possess intermediate frequencies of L3 and high frequencies of E (Table 4). Linia and Kanembou from Chad, Rimaibe from Burkina-Faso, Songhai from Nigeria, and Masalit from Sudan are the only Nilo-Saharan speakers in this class. All the western African countries are represented by different Niger-Congo speaking groups, including the Bateke pygmies from Congo. There are also instances of eastern or southern African Bantu representatives. Finally, Class V includes those samples that present high frequencies for both the L3 and E haplogroups (Table 4). Again, Niger-Congo speakers make up the majority of this class, although Nilo-Saharan from western countries such as Menaka of Mali, Diffa of Niger and Kanuri from Nigeria and those of eastern countries such as Bongor from Chad and Luo from Kenya are included in this class. The two best-represented countries are the western sub-Saharan Africa Nigeria and Cameroon, which provided most of the samples of Niger-Congo speaking individuals. Nevertheless, there are Bantu-specific speakers from Kenya and southern Africa in this class. This group includes the click-speakers Damara from Namibia and South Africa, who genetically have been associated with Bantu-speaking groups rather than to other Khoesan-speaking groups [107].

**Table 3:** AMOVA and k-mean clustering results

Statistic	Variance % within populations	between regions
AMOVA	96.00	4.00
k-2 clustering	43.57	56.43
k-3 clustering	29.68	70.32
k-4 clustering	23.74	76.26
k-5 clustering	19.40	80.60
k-6 clustering	16.75	83.25
k-7 clustering	13.94	86.06

**Table 4:** Frequency values for k-means centroids in 1 to 5 classes

Clase	values L3/E	mt-L3	Y-E
1	low/low	16.9	25.9
2	low/medium	17.6	57.4
3	low/high	17.2	86.6
4	medium/high	36.2	92.9
5	high/high	55.0	87.2

The above results show that the positive correlation found between Y-chromosome haplogroup E and mtDNA haplogroup L3 (and L2) lineages is not strongly associated with either geography nor language. It is better explained as the result of a gradual substitution of the most basal mtDNA (L0, L1, L5) and Y-chromosome (A, B) lineages by the phylogenetically younger clades L2/L3, and E, respectively, throughout Africa. The data also suggest important sex-biased dispersal between populations. These evident gene replacements in Africa have been mainly attributed to recent geographic range expansions of pastoralist and agriculturalist populations from eastern and western Africa at the expense of the hunter-gatherers inhabitants of the Central Africa rainforest [108–110], eastern African forested areas around the Great Lakes [111–114], and the semi-desert open spaces of South Africa [115–118]. Under our hypothesis of an early return to Africa from Eurasia of basal mtDNA L3 and Y-chromosome E lineages and their expansion approximately 70 kya, into East and subsequently West Africa, these lineage replacements must have begun very early. It appears that in this first expansion, mtDNA haplogroup L2 was incorporated by female assimilation, whereas their hypothetical Y-chromosome haplogroup B counterparts were outcompeted by the incoming E chromosomes. An ancient expansion from a Central African source into eastern Africa at 70-50 kya has been associated with haplogroup L2 [119]. Similarly, an early expansion within Africa 60-80 kya involving L3 and possibly L2 was detected long ago [120]. This latter expansion was considered the crucial event in the exit of modern humans from Africa into Eurasia. However, our proposition is that it signaled a backflow from Eurasia and subsequent expansion into Africa.

Moreover, our hypothesis of an early return and subsequent expansion inside Africa of carriers of L3 and E haplogroups might help explain, the Neanderthal introgression detected in the western African Yoruba [121, 122] and in northern African Tunisian Berbers [122].

### A new mtDNA model of the origin and dispersal of *Homo sapiens*

At mtDNA level, data accumulated during the last thirty years, including those contributed by studies of ancient DNA, allow us to propose a more detailed model of the origin and worldwide spread of modern humans than the ones proposed three decades ago. There are three fossil series in northwest, northeast, and southern Africa that chronologically and morphologically recapitulate the evolution of *Homo sapiens* from early archaic humans approximately 600 kya to early modern humans by 200 kya [123]. This ancestral geographic structure is compatible with the higher diversity values observed in African populations compared to the in out-of-Africa populations [124]. The recent dating of Middle Stone Age tools (315 ± 34 kya) and of early modern human fossils (286 ± 32 kya) from Jebel Irhoud in Morocco places the emergence of our species and of the Middle Stone Age, near to each other in time and long before the age of approximately 200 kya previously suggested for the common origin of all humans in eastern Africa [125]. These data coincide in time with the existence of an old Y-chromosome lineage (A00) detected in samples of western-central African ascendance and dated 338 kya (95% CI: 237-581 kya). This age is much older than common estimates based on the Y-chromosome and mtDNA TMRCAs [126]. The fact that the following more divergent Y-chromosome A lineages (A0, A1a) also have a western-central African location, strongly supports this region as the origin of an ancestral human population from which the ancestors of early modern humans emerged [90, 103]. The most ancient splits and expansions of mtDNA lineages are also situated at the hypothetical origin of all extant maternal lineages around this area. Although the earliest L0 clade diverged approximately 145 kya (Additional file 1: Table S3) and had its first expansions in southern Africa (L0d, L0k), the subsequent splits gave rise to L1 and L5 approximately 131 kya and 123 kya, respectively, spreading to western and eastern Africa respectively. These long- range African dispersals place the putative origin of L somewhere in Central Africa (Figure 1a). The same "center-of-gravity" argument has been used by other authors to suggest a Central African origin [127]. While ancestral southern African Khoesan-speaking populations maintain high frequencies of primary L0d and k lineages [94, 106, 128, 129] and while the L1c haplogroup is dominant in the hunter-gatherer populations of central-western Africa [108, 109], L5 in eastern Africa has today only a marginal presence [114, 130]. This marginal presence is likely due to its displacement following incoming waves of better-adapted incomers. The presence of L5 in southern Africa and in eastern Mbuti pygmies [70, 109, 118, 128] is the result of later migrations. It is likely that the next split, approximately 100 kya, also occurred in Central Africa, resulting in sister clusters L2 and L3'4'6 that produced initial westward and eastward expansions, respectively (Fig. 1a). Although the oldest L2 lineages have been sampled in western Africa [131], today, as result of successive expansions within the continent, this clade has a pan-African range [119]. In eastern Africa, the cluster L3'4'6 was the progenitor of the entire Eurasian maternal diversity. Its first lineage was haplogroup L6, which presently is a rare eastern lineage with a deep founder age (about 100 kya) but a rather recent expansion (about 25 kya). It has been found at frequencies below 1% in Egyptians [132], Somalis [133], Kenyans [134], and eastern Nilotes from Uganda [114]. A higher frequency is observed in Ethiopia (3.15 ± 1.15 %) and the maximum frequency (15.8%) is observed in Ongota, an extinguishing linguistic isolate of uncertain adscription [130]. Outside Africa, L6 has not been detected in the Levant [135]. It is present in the Arabian Peninsula at frequencies below 1% in Saudi samples and as high as 12% in some Yemeni samples [136]. Based on the L6 phylogeny (Additional file 2: Figure S1), it appears that not all of the Yemeni lineages are a subset of the eastern African lineages as there is at least one for which its common node coincides with the expansion of the whole haplogroup. Based on its peculiar phylogeography, the possibility that L6 originated from the same out-of-Africa southern migration that colonized Eurasia has been suggested [136]. If this were the case, this early L6 expansion would provide genetic support to the reported presence of modern humans in the Arabian Peninsula, approximately 125 kya based on archaeological evidence [12–14]. This suggestion also receives climatic support as this period coincides with humid environmental conditions in Arabia [137]. However, it appears that this potential human expansion did not extend beyond the peninsula, as L6-derived lineages have not yet been detected across Eurasia. It is likely that the return to arid conditions caused the decline of populations carrying the L6 lineage who had to retreat to refuge areas such as the highlands of Yemen and Ethiopia until more favorable conditions allowed their subsequent recovery in eastern Africa and Yemen. The long mutational stem that precedes the expansion of L6 (Additional file 2: Figure S1), is consistent with such a strong, long-term bottleneck. Subsequent lineage bifurcation produced the ancestors of the L3 and L4 haplogroups (Additional file 2: Figure S1). Presently, the highest frequencies and diversities of L4 are found in eastern Africa, but the haplogroup has spread over the entire continent (Table 3). Furthermore, it has been detected at frequencies below 1% in the Levant [138] and Arabian Peninsula [74, 139]. Some populations show outstanding frequencies of L4 likely due to drift effects. In western Africa, Samoya (28.6%) and Kassena (21.2%) speakers of the Gur linguistic family, show high L4 frequencies [72]. In Ethiopia, samples of the Omotic-speaking Hamer (18.2%), the Cushitic-speaking Daasanach (22.2%), and the Nilotic-speaking Gumuz (24.0%) and Nyangatom (21.6%) also show high frequencies [130, 140].The Tanzanian click-speaking Hadza (58%) and Sandawe (43%) show the highest frequencies of L4 in Africa [111–113]. This latter finding, together with the elevated frequencies of the Y-chromosome haplogroup B-M112 among the Hadza (50%) and Sandawe (15%) [141], suggests that the human expansions from the North were those that most strongly influenced the gene pool of these groups. Considering the age of bifurcation from L3 (around 95 kya), these L4 expansions could have occurred before our proposed return of basal L3 lineages to Africa. However, as the main expansions of its descendant clusters L4a (54.8 kya) and L4b (48.9 kya) [94, 139] occurred around the same time window as those of the majority of the L3 and L2 branches in Africa, the most probable explanation is that improved climatic conditions after 60 kya motivated demographic growth on the African continent. Noticed that the evidence of a first expansion of L3 into East Africa [89] is likewise in support of the out-of-Africa scenario than of an Eurasian back-flow as proposed here. We hypothetically situated the L3'4 node in northeast Africa or the Near East (Fig. 1a) to allow an out-of-Africa expansion of the pre-L3 clade. The Y-chromosome CDEF ancestor must be this clade's male counterpart. Other female and male lineages might have moved with them; if so, they presumably, became extinct without contributing to either the maternal or paternal gene pools of the living human populations of Eurasia.

Under the scenario proposed here, early anatomically modern humans traveled out of Africa approximately 125 kya with a simple Middle Stone Age technology that was not superior to that manufactured by the Neanderthals. Favored by mild climatic conditions, these African pioneers progressed through West Asia and further followed the southern boundary of Central Asia, potentially overlapping en route the southern geographic range occupied by the now extinct hominins. Furthermore, as Neanderthals and early modern humans are associated with the same lithic industries in the Levant, it seems plausible that a new vision of the Early- and Mid-Middle Paleolithic fossil and archaeological records of those regions [87, 142, 143] might uncover the path followed by those early African colonizers. Under favorable conditions for both hominin groups, we might predict limited exchange of skills, lithic technology, and sex. However, when glacial environments became dominant after 75 kya, Neanderthals retreated southwards, possibly displacing humans as they went. Confronted with the northern foothills of the Himalayas, humans moved in two directions: westwards, eventually to return to Africa; and eastwards, eventually reaching southeastern Asia via China (Fig. 1b). The second part of this model has been outlined in preceding articles [53–55].

## Additional files

Additional file 1: **Table S1**. Complete mtDNA macrohaplogroup L sequences. Table S2. Frequencies of mtDNA haplogroups L2 and L3 and Y-chromosome haplogroup E lineages across Africa. Table S3. Coalescence ages in thousand years (kya) with 95% coefficient intervals (CI), or standard deviations, for the main mitochondrial DNA African haplogroups. Table S4. Coalescence ages in thousand years (kya) with 95% coefficient intervals (CI), or standard deviations, for Y-chromosome most recent common ancestor (MRCA), the out-of-Africa event, and the splits of haplogroup DE and E. Table S5. Population clustering into five classes. Table S6. k-means cluster results using African populations characterized by mtDNA L3 and Y-chromosome E haplogroup frequencies. Table S7. k-means cluster results using African populations characterized by mtDNA L2 and L3 and Y-chromosome E haplogroup frequencies. (XLSX 403 kb)

Additional file 2: **Figure S1**. Phylogenetic tree of mtDNA macrohaplogroup L complete African sequences produced in this study. (XLSX 71 kb)
